# Deactivation of the dorsal anterior cingulate cortex indicated low postoperative sports levels in presurgical patients with chronic ankle instability

**DOI:** 10.1186/s13102-021-00353-6

**Published:** 2021-10-09

**Authors:** Xiao’ao Xue, Shengkun Li, Hongyun Li, Qianru Li, Yinghui Hua

**Affiliations:** Department of Sports Medicine, Huashan Hospital, Fudan University, 12 Wulumuqi Middle Road, Shanghai, 200040 China

## Abstract

**Background:**

Injury-related fear contributed to disability in chronic ankle instability (CAI), while there still lacked exploration on the appraisal processes of the injury-related stimuli. This study aimed to compare the neural activities of the appraisal processes of sprain-related stimuli between presurgical chronic ankle instability patients and healthy controls through functional magnetic resonance imaging (fMRI) and evaluate its relationships with the clinical outcomes of orthopedic surgeries.

**Methods:**

Eighteen presurgical CAI patients and fourteen healthy controls were recruited and underwent an fMRI session with visual stimulation of movies that showing typical ankle sprains accidents or control videos and the corresponding fear ratings. The clinical outcomes were collected at baseline and a minimum of 2 years after surgery; these included the American Orthopaedic Foot and Ankle Society (AOFAS) scores, the Numeric Rating Scale (NRS) scores, and the Tegner Activity Rating Scale scores. The two-sample t-test would be applied to identify which brain regions were influenced by CAI, and the correlation analysis would be applied to measure the relationship between the activation and clinical outcomes.

**Results:**

Dorsal anterior cingulate cortex (dACC) was deactivated in CAI patients when compared with healthy controls, and the dACC deactivation strength revealed a moderate correlation with the values of fear ratings for all participants. The deactivation strength was negatively correlated with AOFAS at baseline, with Tegner at follow-up and its improvement.

**Conclusions:**

Presurgical CAI patients presented deactivated dACC as a different neural activity of appraisal processes of sprain-related stimuli when compared with healthy controls, which was associated with lower postoperative sports levels. More comprehensive patients care including psychological interventions were needed in the clinical management of chronic ankle instability.

**Supplementary Information:**

The online version contains supplementary material available at 10.1186/s13102-021-00353-6.

## Background

The lateral ankle sprain is one of the most common sport-related injuries [1]. Over 2 million ankle sprains are treated in the US and UK each year, resulting in about $2 billion of health-care costs [2]. In the long-term, more than 30% of patients report repetitive bouts of ankle giving way and recurring sprains, termed as chronic ankle instability (CAI) [3]. Surgical management is usually suggested to treat patients who suffered serious symptoms and failed in conservative treatments to fix mechanical structures, however, some patients do not recover and fail to return to practice sports [4]. Many researchers have focused on surgical techniques and postoperative rehabilitation [5–7]. Although psychological factors have been recognized as important mediators to recovery, little is known about how they would affect clinical outcomes in patients with CAI [8].

Injury-related fear has been proposed to be one of the reasons for disability in musculoskeletal injuries [8–10]. Previous studies have suggested that patients with CAI have greater injury-related fear than healthy people, which may also lead to avoidance coping responses, such as declines in sports [8, 10, 11]. Furthermore, for the patients who had undergone surgical treatments, the appraisal of injury-related stimuli and the subsequent choices of coping strategy might also play an important role in postoperative outcomes [11–14]. According to studies on anterior cruciate ligament reconstruction, patients with a positive appraisal of the injury report a better postoperative joint function and need less time to return to practice sports compared to those patients with a passive one [15]. For example, when comparing the presurgical patients who accepted the fact of their injuries and positively participated in postoperative rehabilitation programs, and those who stuck with the fear of injuries and avoided any movements, the former ones were more likely to have less postoperative joint adhesion and muscle loss [16]. Similar research in patients with CAI is lacking, especially for the presurgical patients who suffered the influences of ankle sprains more seriously. The appraisal processes of sprain-related stimuli could be fatal for the presurgical CAI patients to get better clinical outcomes during the postoperative rehabilitation stage. Therefore, the present study is aiming at exploring the appraisal processes of sprain-related stimuli and their effect on the postoperative clinical outcomes in presurgical patients with CAI.

Functional magnetic resonance imaging (fMRI) has been widely used in psychology and neuroscience to evaluate the brain’s activations in relation to human behavior [17]. As for the principle of fMRI, briefly, when a brain area is performing its function, the automatic blood regulation will surpass the oxygen consumption and decrease deoxyhemoglobin concentration, so that the localization and quantification of activated areas can be acquired by increased T2 signal, named blood oxygenation level-dependent (BOLD) signal [18]. In the present study, BOLD signal strength will be recorded as the neural activities of appraisal processes of sprain-related stimuli during the task (with visual stimulation of movies showing typical ankle sprains accidents or control videos) for the presurgical CAI patients who suffered from ankle sprains, which could be important in terms of developing more comprehensive patient care and improve the outcomes of orthopedic surgeries [15, 19–21].

Therefore, the present study had two main objectives: (1) investigating the neural activities of appraisal processes of sprain-related stimuli in presurgical CAI patients compared with healthy people, as a cross-sectional design; (2) analyzing the relationships between the central appraisal activities caused by sprain-related stimuli and postoperative outcomes in patients with CAI, as a case series design. We hypothesized that there are different neural activities of the appraisal process to the sprain-related stimuli between presurgical CAI patients and healthy people, and the strength of it would influence the surgical outcomes of patients with CAI.

## Methods

The present study employed a cross-sectional fMRI design and a prospective cohort design following-up the patients’ group with a minimum of 2 years postoperative. The article was composed according to the publishing guidelines of The Strengthening the Reporting of Observational Studies in Epidemiology (STROBE) statement [22]. This study has been approved by the appropriate ethics committee (Institutional Review Committee of Huashan Hospital) and has therefore been performed in accordance with the ethical standards laid down in the 1964 Declaration of Helsinki.

### Participants

Between December 2016 and June 2017, 32 right-footed participants (for the consistency of functional asymmetry of brain) were recruited to participate in an fMRI study. These included 18 presurgical patients who suffered from chronic ankle instability (11 males, aged 29.0 ± 7.2 years; range, 17–42) and 14 healthy controls from the general community without any lower limb injury history (10 males, aged 30.6 ± 6.5 years; range, 23–46). The patients were further followed at a minimum of 2 years after the orthopedic surgery. All participants signed written informed consent before participation.

Inclusion criteria for presurgical patients with CAI were: (1) persistent symptoms of pain, ankle giving way, and repetitive inversion sprains after 3 to 6 months of nonsurgical treatment; (2) positive Anterior Drawer Test and/or Talar Tilt Test; (3) unilaterally impaired lateral ankle ligaments, including the anterior talofibular ligament and/or the calcaneofibular ligament, confirmed by preoperative MRI [7, 23]. All the patients were planned to accept the surgery. Exclusion criteria for both groups were as follows: (1) history of other musculoskeletal disorders or surgeries to the lower extremities; (2) history of cardiovascular, respiratory, neurological, autoimmune, or other major medical illnesses; (3) current usage of pharmaceuticals such as psychotropics, excitants, or beta-blockers; (4) people who played basketball on a regular basis due to the content of fMRI task (videos of basketball players).

### Part 1: task design and data analysis of fMRI

**Fig. 1:**
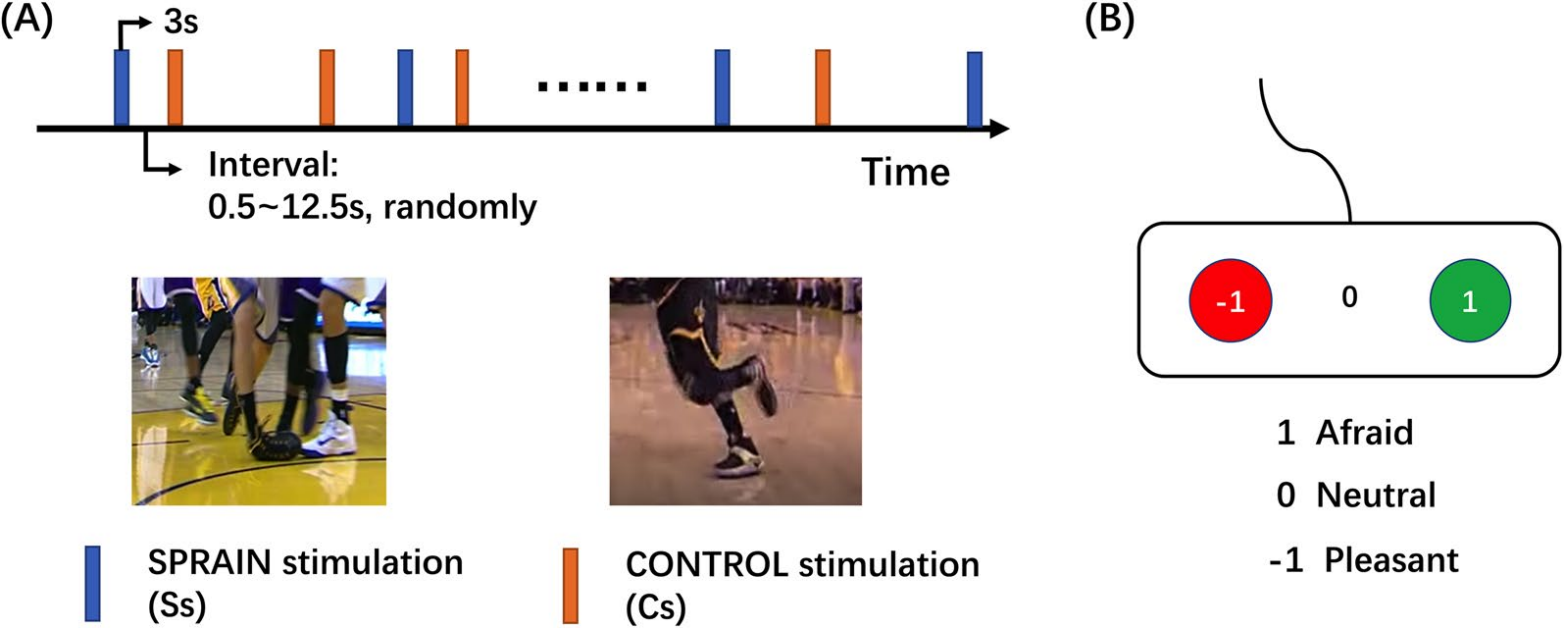
**a** Each run was composed of 8 SPRAIN stimuli of sprain accident and 8 CONTROL stimuli of basketball playing presented in a pseudorandom manner; **b** An MR-compatible assessment responder with three rating options: afraid (− 1), neural (0, no response) or pleasant (+ 1), to measure the degree of fear induced by each stimulus

On the day before surgery, an fMRI test was performed for both groups as the first part of this study. The task consisted of the event-designed visual stimuli that were applied to simulate the neural activities of coping response in relation to the injury-related fear [24]. Sprain stimuli (Ss) consisted of self-edited 3 s-long videos of fearful sprain accidents in basketball games with a third-person view, control stimuli (Cs) consisted of matched videos without sprains [25, 26]; all videos were presented to the participants. (Fig. 1a) The intervals between stimuli were randomly varied between 0.5 s and 12.5 s [27]. Each participant performed three runs repeatedly, and each run was composed of 8 Ss and 8 Cs that presented in a pseudorandom manner (the orders of the stimuli were generated randomly before the study and kept the same for all participants). During each stimulus, participants were asked to imagine how they would feel if that situation occurred to them and then rate their feelings immediately through an MR-compatible assessment responder. There were three rating options: afraid (− 1), neutral (0, no response) or pleasant (+ 1) so that the fear levels induced by two kinds of stimuli could be evaluated by the average rating scores, and the levels of injury-related fear could be calculated by difference values quantitatively [28] (Fig. 1b).

A 3.0-T Siemens Magnetom Verio scanner equipped with an 8-channel head coil was used for scanning. Detailed data acquisition and image preprocessing steps are provided in the Additional file 1. For the first-level statistical analysis, Statistical Parametric Mapping (SPM8, Department of Cognitive Neurology, London) that implemented in MATLAB version R2014a (The MathWorks Inc., Natick, Massachusetts) was used. A general linear model was specified by SPM8 for the Ss conditions, Cs conditions, and rest conditions according to time series. BOLD signal strength (beta value) of each condition were obtained, convolved with the canonical hemodynamic response function and high-pass filtered (1/128 Hz) to remove low-frequency noise caused by scanner signal drifts [29]. For the second-level statistical analysis, Restplus Version 1.22 (http://restfmri.net/forum/index.php?q=rest) was used. Contrast images for “Ss condition > rest condition”, “Cs condition > rest condition” and “Ss condition > Cs condition” for each participant were calculated to reveal neural activations caused by the two kinds of stimulations and the sprain-related stimuli without basketball playing scene. The contrast images of “Ss condition > Cs condition” were compared between groups using a two-sample t-test to identify which brain regions had significant differences in neural activations of appraisal processes of the visual presented sprain-related stimuli. The multivariable regression was also repeated to control age and sex. The two-tailed cluster-level threshold of *p* < 0.05 and voxel-level threshold of *p* < 0.001 with Gaussian Random Field corrections were used to define significance.

The areas showing significant differences were saved as masks and the signal strength within them was extracted from the contrast images of “Ss condition > Cs condition” for each participant to perform further analyses. If the signal strength was negative, a positive–negative switch would be displayed and defined as deactivation strength to facilitate analysis and discussion.

### Part 2: treatment procedure and clinical evaluation

All patients were treated in our hospital, and the arthroscopic ankle stabilization surgeries were performed by a senior surgeon (the corresponding author) according to previously described standardized methods [5, 30]. Briefly, the lateral ligament repair would be considered if the ATFL remnant was sufficient and in good condition, or anatomical reconstruction with semitendinosus allograft or gracilis autograft would be performed [5, 30]. All patients were encouraged to walk without support if they were able to, and they all accepted standardized postoperative rehabilitation after the operation. For the second part of this study, patients were followed-up at a minimum of 2 years after surgery, which was long enough for them to get satisfactory postoperative outcomes according to our previous studies. Clinical evaluations, including (1) the 100-points American Orthopaedic Foot and Ankle Society (AOFAS) score for ankle function, which measured the severity and frequency of pain (40 points), activity limitations/support requirement (10 points), maximum walking distance (5 points), walking surfaces (5 points), gait abnormality (8 points), sagittal motion (8 points), hindfoot motion (6 points), ankle-hindfoot stability (8 points), and alignment of ankle-hindfoot (10 points); (2) the 10-point Numeric Rating Scale (NRS) for subjective severity of current pain, from “no pain” (0 points) to “unbearable pain” (10 points); (3) and the 10-point Tegner Activity Rating Scale (Tegner) score for sports level, form “sick leave or disability pension because of the joint problem” (0 points) to “Competitive sports Soccer-national and international elite” (10 points), were administered at baseline for both groups and re-administered for the patient’s group. Improvements in scores would be calculated as the difference in values between the clinical scores at the two-time points. The time of return to work after surgery was also measured. All evaluations were performed by a blinded physician.

### Statistical analysis

Statistical analysis was performed in Graphpad Prism Version 8.0 (GraphPad Software, San Diego, California). Descriptive statistics were calculated as mean ± standard deviation. Mann–Whitney U tests, Chi2 tests, and 2-sample t-tests were performed to compare demographic variables between groups. Paired t-tests or Wilcoxon tests were performed to compare clinical scores at baseline and follow-up in the patient’s group. The fear ratings under each condition were analyzed by group-level ANOVA with pair-wise Bonferroni multiple comparison tests. Correlations between signal strength and clinical scores were determined by Pearson or Spearman correlation coefficients. The selections of parametric or non-parametric tests were based on variance homogeneity and normality of the data. The statistical significance level was set at 0.05. Participants with excessive head motions (more than 3 mm or 3°) in the first part and patients who dropped out from the follow-up or reinjured in the second part were excluded [31].

## Results

### Demographic and clinical features

A total of 13 healthy controls and 16 presurgical CAI patients were enrolled in the first part of this study (1 healthy control and 2 patients were excluded due to excessive head motion). Among the patients, 11 underwent the repair and 5 underwent the reconstruction (2 with allograft and 3 with autograft) of the lateral ligaments. There were no significant differences between the two groups in all demographic features (*p* > 0.05), while significant differences were found in the AOFAS, NRS and Tegner score (*p* < 0.01) between two groups, indicating worse ankle functions, pain levels and sports participation in the patient group at baseline (Table 1).

**Table 1:** Demographic variables of control group and patient group

	Control group (n = 13)		Patients group (n = 16)		*p* value
	Mean	SD	Mean	SD	
Age (years)	29.5	5.1	28.6	6.8	0.71
Weight (kg)	68.6	11.9	62.7	10.1	0.17
Height (m)	1.73	0.08	1.70	0.10	0.41
Education level (years)	16.0	1.2	15.0	2.2	0.15
Male/female	10/3		10/6		0.45
Duration (months)	–	–	37.5	33.9	–
AOFAS	100.0	0.0	73.7	10.8	< 0.01
NRS	0.0	0.0	3.3	1.5	< 0.01
Tegner	6.2	0.6	3.8	1.0	< 0.01

AOFAS, American Orthopaedic Foot and Ankle Society score; NRS, Numeric Rating Scale; Tegner, Tegner Activity Rating Scale

### Part 1: fMRI tests on the central Appraisal response to the fear

**Fig. 2:**
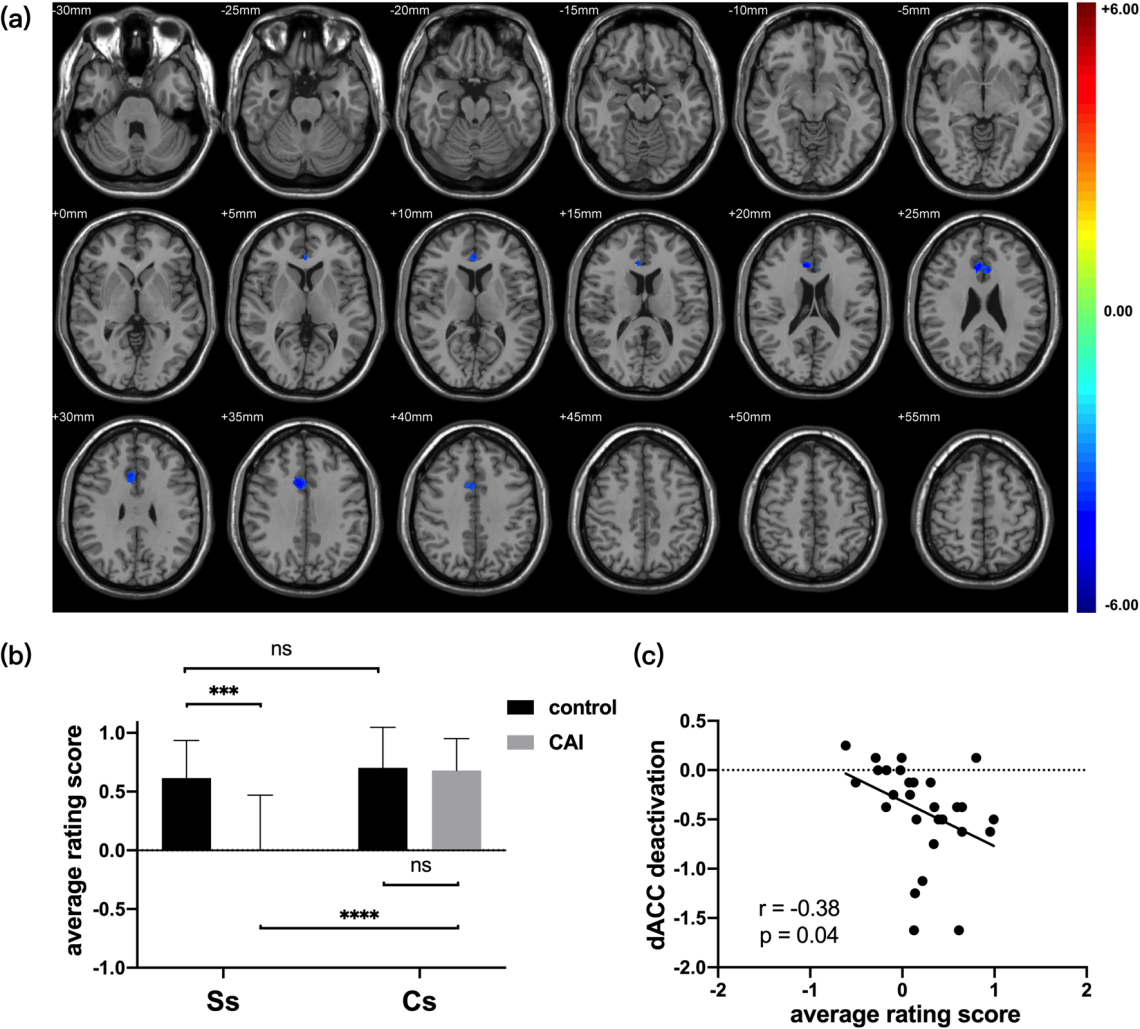
**a** The result of two sample t test of contrast images of “Ss condition > Cs condition” (SPRAIN stimuli minus CONTROL stimuli) between Patient Group and Control Group after two-tailed Gaussian Random Field correction with the cluster-level threshold of *p* < 0.05 and voxel-level threshold of *p* < 0.001; **b** the comparison of average rating scores between two groups under two stimuli after pair-wise Bonferroni multiple comparison tests; **c** the correlation between dACC deactivation strength and difference values of fear ratings; ns, no significant difference;*adjusted *p* < 0.05; **adjusted *p* < 0.01; ***adjusted *p* < 0.001; ****adjusted *p* < 0.0001

For the fMRI evaluation, one cluster within “Ss condition > Cs condition” contrast was significantly deactivated in patients with CAI when compared with healthy controls. The cluster was located in Brodmann’s area 24 and 32, defined as the dorsal anterior cingulate cortex (dACC). The size of the cluster was 350 voxels, the peak MNI coordinate was (8, 26, 24), and the intensity (t value) was − 4.58. (Fig. 2a). A positive–negative switch was performed to the signal strength of “Ss condition > Cs condition” contrast images as the deactivation strength. When comparing fear rating between groups, no significant difference under Cs (*p* > 0.99, 95% CI − 0.61 to 0.41) and greater fear levels in patients under Ss (*p* < 0.01, 95% CI 0.23 to 1.00) were observed. (Fig. 2b) The correlation between dACC deactivation strength and the difference in values of fear ratings (between Ss and Cs) revealed a moderate correlation (*p* = 0.04, r = − 0.381, 95% − 0.66 to − 0.02) for all participants (Fig. 2c).

### Part 2: relationships between fMRI signals and clinical outcomes

Median follow-up time was 34 (ranged from 30 to 35) months for the patients in the second part of this study. One patient (reconstruction with autograft) who dropped out from follow-up and two patients (repair) who suffered from another ankle sprain accident after the surgery were excluded. None of the remaining patients had further ankle sprains or other orthopedic injuries. All clinical outcomes, including AOFAS (*p* < 0.01), NRS (*p* < 0.01), and Tegner scores (*p* < 0.01), were significantly improved at follow-up. The average time to return to work was 7.0 ± 1.7 weeks.

To investigate the relationship between dACC deactivation strength and durations, time to return to work, clinical scores at baseline and follow-up, and the improvements of clinical scores, a set of correlation analyses were performed. At the baseline, AOFAS was significantly negatively correlated with the deactivation strength (r = − 0.60, *p* = 0.03). At follow-up, the postoperative Tegner score (r = − 0.59, *p* = 0.04) was found to be significantly negatively correlated with deactivation strength. For the improvement, the deactivation strength was shown to be significantly correlated (positively) with the AOFAS (r = 0.80, *p* < 0.01) and (negatively) with the Tegner improvement (r = − 0.66, *p* = 0.02). A trend of correlation between deactivation strength and time to return to work was also observed (r = 0.55, *p* = 0.05). No other significant correlations were identified (*p* > 0.05), (Table 2).

**Table 2:** Correlations of dACC deactivation strength with clinical outcomes in patients of baseline, 3 years follow-up and their improvements

dACC deactivation	Baseline			Follow-up			Improvement		
	r	95% CI	*p*	r	95% CI	*p*	r	95% CI	*p*
AOFAS^a^	− 0.60*	− 0.87 to − 0.08	0.03	0.04	− 0.52 to 0.58	0.89	0.80**	0.44 to 0.94	< 0.01
NRS^b^	0.33	− 0.29 to 0.75	0.28	− 0.04	− 0.59 to 0.54	0.90	− 0.33	− 0.76 to 0.28	0.27
Tegner^b^	− 0.14	− 0.65 to 0.46	0.65	− 0.59*	− 0.86 to − 0.03	0.04	− 0.66*	− 0.89 to − 0.15	0.02
Return to work (weeks)^a^	–			0.55^^^	0.002 to 0.85	0.05	–		
Duration^a^	− 0.10	− 0.61 to 0.48	0.76	–			–		

dACC, Dorsal Anterior Cingulate Cortex; AOFAS, American Orthopaedic Foot and Ankle Society score; NRS, Numeric Rating Scale; Tegner, Tegner Activity Rating Scale

^a^Pearson correlation coefficient

^b^Spearman correlation coefficient

^^^*p* = 0.05; *p < 0.05; **p < 0.01

## Discussion

The most important finding of this study was the deactivation of dACC as a different appraisal process of the sprain-related stimuli in presurgical patients with CAI when compared with healthy people, which was also associated with lower postoperative sports level. To the best of our knowledge, the present study is the first fMRI study on the evaluation of appraisal processes of the sprain-related stimuli and its relationship with surgical outcomes in patients with CAI.

Numerous studies have demonstrated the role that injury-related fear plays in the disability of musculoskeletal diseases [8, 10, 32–36]. However, all of the past and recent studies on injury-related fear in patients with CAI have used self-reported evaluation of fear and avoidance and lacked a further exploration of the neural activities of its appraisal processes. To overcome this limitation and advance the knowledge in this area the present study used fMRI to explore the neural activities of its appraisal processes of the injury-related stimuli quantitatively [17]. Comparing the whole brain activation, a significant deactivation within dACC was observed under visual presented sprain-related stimuli in patients with CAI compared with healthy controls, in this study.

The dorsal anterior cingulate cortex is a part of the limbic system located in the inner side of the cerebral hemispheres and associated with the appraisal of fear-related stimuli [24, 37]. However, increased activity of dACC has commonly been observed in previous studies on fear emotion, which seemed to be contrary to our results [28, 38]. The explanation for that may be due to differences in the study designs. Previous studies aimed to evaluate the expression of fear, and therefore, stimuli with low cognitive load (such as fearful pictures) were presented to induce conditioned fear, leading to increased activation of the dACC [24, 38]. In the present study, we aimed to simulate the appraisal process of the sprain accidents, and therefore, self-imaginations with high cognitive load were induced when watching the sprain accident videos, which may have resulted in the deactivation of the dACC [25]. A previous study on shoulder apprehension with a similar task design to the present study also observed the deactivation of the dACC [25]. In addition, regarding its positive correlation with fear levels, this change may represent a passive appraisal process of sprain-related stimuli and be associated with injury-related fear of the patient with CAI [11, 21, 39].

To further explore the clinical meanings of this appraisal process in patients with CAI, the relationship between dACC deactivation strength and clinical outcomes were evaluated. The significant correlation with the AOFAS score at baseline suggested that worse ankle function was associated with a higher passive appraisal process of sprain-related stimuli. The experience of serious symptoms and recurrent sprains in patients might increase their appraised degrees of the threat caused by ankle sprains, induced more passive emotions (e.g. injury-related fear) and the corresponding avoidance coping of movements (especially the rehabilitation training), which could also make their ankle functions worse [11, 14, 40]. Furthermore, this change might depend more on severity than the time length of CAI, as supported by its non-significant correlation with the duration of symptoms. At a minimum follow-up of 2 years, the significant correlations between dACC deactivation and postoperative Tegner scores and its improvement fitted the previous evidence that the passive appraisal process and its corresponding avoidance coping response would become maladaptive in the long term recovery, making patients turning away from risky behaviors to avoid their fear of injuries (such as postoperative rehabilitation) [15, 21, 39]. A trend of correlation with the time to return to work also supported this view, as returning to sports or heavy physical activities might not be a priority for patients from community anymore, differently in comparison to previous studies on athletes who lived on sports [12, 13, 41]. Although in contrast with previous findings, we observed that patients with higher dACC deactivation reported a higher improvement in ankle function [15, 21]. A potential reason for that, as supported by the Tegner scores could be that the avoidance of heavy physical activity facilitated a reduction in residual symptoms postoperatively. Although previous studies indicated that dACC contributed to pain processing, the non-significant result in our study suggested that the deactivated dACC might not play an important role in pain among patients with CAI in this study [42].

We intended to investigate the mechanisms underpinning the appraisal processes of the injury-related stimuli in this study, because our clinical experience suggested that some postoperative patients, were extremely worried about the condition of their ankle and preferred to avoid movement after ankle orthopedic surgeries. Our findings provide further insight into the appraisal processes of the injury-related stimuli in patients with CAI and emphasized its important role in both progression and post-operation stages. This in turn might shift the attention to psychological factors and, hopefully, promote the development of more specific psychological interventions on passive appraisal processes of the injury-related stimuli in order to improve the surgical outcomes for patients with CAI and even other sports injuries in the future.

This study was not without limitations. First, the sample size was relatively small, however, it was similar to other studies with similar protocol [25, 28]. More multivariable regressions or subgroup analyses in a larger sample might explain the role of appraisal processes of the sprain-related stimuli in presurgical patients with CAI better. Next, the current study only performed the fMRI tests at baseline due to the difficulty in recalling patients back from all over the country. A more detailed longitudinal study on how the surgery (e.g. repair or reconstruction) or rehabilitation influenced dACC activation should be conducted in future studies. Then, this study only focused on the fMRI test of neural activities with fear rating, and the lack of standardized self-reported questionnaires on the emotional responses and coping procedure of the CAI patients before/after the surgery did reduce the reliability of this study. Finally, the patients enrolled in this study were not fully in line with the recommendation of the International Ankle Consortium. However, as this study focused on the surgical outcomes, our inclusion may represent the presurgical patients with serious symptoms and mechanical instability better than the recommended one.

## Conclusions

Our study suggested that presurgical CAI patients presented deactivated dACC as a different neural activity of appraisal processes of on sprain-related stimuli when compared with healthy controls, and the higher deactivation strength was associated with lower postoperative sports levels. As the psychological factors might influence the outcome of orthopedic surgeries, more comprehensive patients cares including psychological interventions are needed in the clinical management of presurgical patients with CAI.

## Supplementary Information

**Additional file 1.** Detailed fMRI data acquisition and preprocessing steps.

## Data Availability

The original imaging data used to support the findings of this study have not been made available because of the patient privacy, while the extracted data and the mask of the significant cluster are available from the corresponding author upon request.

## Abbreviations

CAI: Chronic ankle instability
dACC: Dorsal Anterior Cingulate Cortex
fMRI: Functional magnetic resonance imaging
BOLD: Blood oxygenation level-dependent
AOFAS: The American Orthopaedic Foot and Ankle Society scores
NRS: The Numeric Rating Scale scores
Ss: Sprain stimuli
Cs: Control stimuli
