# Supplementary material for: Deactivation of the dorsal anterior cingulate cortex indicated low postoperative sports levels in presurgical patients with chronic ankle instability
Source: BMC Sports Sci Med Rehabil. 2021 Oct 9;13:121. doi: 10.1186/s13102-021-00353-6 (PMC8501719; doi:10.1186/s13102-021-00353-6)
Supplement: Supplementary file 1 — Additional file 1. Detailed fMRI data acquisition and preprocessing steps. [file 13102_2021_353_MOESM1_ESM.docx]

***fMRI Data ﻿Acquisition.*** A 3.0-T Siemens Magnetom Verio scanner equipped with an 8-channel head coil was used to obtain the images. During the scanning, participants were lying supine in the scanner with their head fixed by a foam pad to minimize their head movement in the head coil. For structural 3D T1 imaging, we used a fast spoil gradient recall sequence as follows: ﻿matrix size = 256*256, repetition time (TR) = 2300 ms, echo time (TE) = 2.98 ms, flip angle(FA) = 9°, field of view (FOV) = 256*256 ﻿mm^2^, and slice thickness = 1 mm. For functional imaging, we used a T2*-weighted echo-planar image (EPI) sequence as follows: matrix size = 64*64, TR = 2200 ms, TE = 35 ms, FA = 90°, FOV = 224*224 ﻿mm^2^, slice thickness = 3.5 mm, and ﻿number of acquisitions = 178.

***fMRI Data Preprocessing***

Statistical Parametric Mapping (SPM8, Department of Cognitive Neurology, London) implemented in MATLAB version R2014a (The MathWorks Inc., Natick, Massachusetts) was used.﻿ The first 3 time points were removed for signal equilibrium. The slice timing procedure was applied to correct for the different acquisition times of each slice. The interscan head motion was corrected by realigning the functional images to the first image. The mean EPI image was produced after realignment. The mean EPI image was then used for the co-registration of individual T1-weighted, 3D structural images. A unified segmentation algorithm was used to segment the co-registered structural image into gray matter (GM), white matter (WM) and cerebrospinal fluid (CSF). ﻿The functional images were then spatially registered to the Montreal Neurological Institute (MNI) space (resampled to 2*2*2 mm^3^) using the normalization parameters estimated during unified segmentation. ﻿The registered images were spatially smoothed with a Gaussian kernel of 8 mm full-width half-maximum (FWHM). ﻿
